# Quantitative Label-Free Proteomic Analysis of Milk Fat Globule Membrane in Donkey and Human Milk

**DOI:** 10.3389/fnut.2021.670099

**Published:** 2021-06-22

**Authors:** Xinhao Zhang, Bo Jiang, Chuanliang Ji, Haijing Li, Li Yang, Guimiao Jiang, Yantao Wang, Guangyuan Liu, Guiqin Liu, Lingjiang Min, Fuwei Zhao

**Affiliations:** ^1^College of Animal Science and Technology, Shandong Agricultural University, Taian, China; ^2^National Engineering Research Center for Gelatin-Based Traditional Chinese Medicine, Dong-E E-Jiao Co., Ltd, Liaocheng, China; ^3^Institute of Animal Husbandry and Veterinary Medicine, Beijing Academy of Agricultural and Forestry Sciences, Beijing, China; ^4^Shandong Donkey Industry, Technology Collaborative Innovation Center, Liaocheng University, Liaocheng, China; ^5^College of Animal Science and Technology, Qingdao Agricultural University, Qingdao, China

**Keywords:** human, donkey, quantitative proteomic, comparison, milk fat globule membrane

## Abstract

Previous studies have found donkey milk (DM) has the similar compositions with human milk (HM) and could be used as a potential hypoallergenic replacement diet for babies suffering from cow's milk allergy. Milk fat globule membrane (MFGM) proteins are involved in many biological functions, behaving as important indicators of the nutritional quality of milk. In this study, we used label-free proteomics to quantify the differentially expressed MFGM proteins (DEP) between DM (in 4–5 months of lactation) and HM (in 6–8 months of lactation). In total, 293 DEP were found in these two groups. Gene Ontology (GO) enrichment analysis revealed that the majority of DEP participated in regulation of immune system process, membrane invagination and lymphocyte activation. Several significant Kyoto Encyclopedia of Genes and Genomes (KEGG) pathways were determined for the DEP, such as lysosome, galactose metabolism and peroxisome proliferator-activated receptor (PPAR) signaling pathway. Our study may provide valuable information in the composition of MFGM proteins in DM and HM, and expand our knowledge of different biological functions between DM and HM.

## Introduction

Donkey milk is more similar to human milk because of its total protein and lactose contents, similar fatty acid and protein profiles (1). It has been indicated to be more suitable for children and elderly people due to its remarkable nutritional value and less allergenic. Besides, milk fat globules in DM are smaller and more easily digested and absorbed by infants (2). With its obvious advantages, the demand of direct consumption for DM has increased.

Milk fat globule membrane (MFGM), a three-layer membrane, covers on the surface of the milk fat globule (3). MFGM proteins make up only 1–2% of the total milk proteins, but they are thought to play important roles in biological processes, including cell growth promotion, cell activity regulation and defense mechanisms against bacteria and viruses in infants (4). Investigations on MFGM proteome have primarily focused on profiling analyses of MFGM fractions from different mammals. For example, Yang et al. identified 232 differentially expressed MFGM proteins in HM and CM across different lactation stages using the iTRAQ proteomic approach (5). To reveal the differences in the formation of MFGM in different mammals, the MFGM proteins of cow, yak, buffalo, goat, horse, camel and human were also compared by iTRAQ proteomics (6). Recently, Li et al. hoped to explore the changes in the regulation mechanism of different lactation stages by analyzing the differences of MFGM proteins between donkey colostrum and mature milk (2). However, studies of the DM MFGM proteome are relatively sparse and less comprehensive, especially fully comparative analyses of MFGM protein compositions and potential biological activities between DM and other species.

The aim of our study was to compare the expression of MFGM proteins between DM and HM by label-free quantification and to explore the biological processes they were involved in. The results are helpful for us to better understand the differences between DM and HM in the composition of MFGM and provide strong support for the future development of formula milk using donkey milk as nutritional provider.

## Materials and Methods

### Sample Collection and Treatment

HM samples were donated by twelve healthy mothers with the lactation of 6–8 months with written informed consent which indicated that the milk would be used in research. Twelve DM samples were obtained from a local farm breeding Dezhou donkeys (6–9 years old) with the lactation of 4–5 months in Liaocheng City of Shandong province, China. All procedures involving donkeys were performed by the Shandong Agricultural University Animal Care and Use Committee (approval number, SDAUA-2020-053). Four samples of each group were randomly mixed and stored at −80°C.

MFGM proteins separation was done as described by previous report (2). All samples were centrifuged at 4°C and 10,000 g for 15 min to obtain the upper fat portion containing MFGM. The upper fat layer was washed with 10 ml cold phosphate-buffered saline (0.24 g KH2PO4, 1.44 g Na2HPO4, 8 g NaCl and 0.2 g KCl were dissolved in 800 ml deionized water, the PH was adjusted to 7.4 and the volume was fixed to 1,000 mL) and homogenized by ultrasound (80 W, 15 s) for 3 times. The mixture was centrifuged at 4°C and 10,000 g for 1 h. Then, acetone was added to the collected supernatant to segregate the fat globules overnight. After centrifugation at 15,000 g at 4°C for 30 min, the supernatant was poured away. Finally, lysate was added to the protein particle sample and ultrasonicated for 15 s at 80 W for 10 cycles.

### MFGM Protein Digestion

Thirty microliters protein solution were taken from each sample and dithiothreitol (DTT) were added to the final concentration of 100 mM. The samples were incubated in boiling water for 5 min and cooled to room temperature. Then, 200 μL UA buffer (8 M urea, 150 mM TrisHCl, pH 8.0) was added and mixed thoroughly. After that, each sample was transferred into a 10 kDa ultrafiltration centrifuge tube (Sartorius, Germany) and centrifuged at 14,000g for 15 min, then discarded the filtrate (repeat this step once). 100 μL IAA buffer (100 mM IAA in UA) was added to the protein mixture in the tube and shaken at 600 rpm for 1 min. The mixture was left at room temperature in dark for 30 min and centrifuged at 14,000 g for 15 min and the process was repeated twice. Hundred microliters 25mm NH4HCO3 solution was added to the tube and centrifuged at 14,000 g for 15min and repeated the procedure twice. Subsequently, 40 μL trypsin buffer (4 μg trypsin in 40 μL 100mM NH4HCO3) were added to the mixture and sample was shaken at 600 rpm for 1 min. The mixture was left at 37°C for 16–18 h and then transferred to a new collecting tube and centrifuged at 14,000 g for 15min. Then, 40 μL 25 mm NH4HCO3 was added in the mixture and sample was centrifuged at 14,000 g for 15 min to collect the filtrate. The peptide was desalted by C18 cartridge (Sigma, USA). After freeze-drying, the peptide was dissolved in 40 μL 0.1% formic acid solution.

### LC- MS/MS Experiments

Each sample was separated by the Easy-nLC^TM^ (Proxeon Biosystems, Thermo Fisher Scientific) orbitrap HPLC system with nano-flow. Buffer A was 0.1% formic acid, and buffer B was 84% acetonitrile in 0.1% formic acid. The chromatographic column was equilibrated with 95% buffer A. The sample was loaded by an automatic sampler to the sample column (Thermo Scientific Acclaim Pepmap 100, 100 μm × 2 cm, nanoViper C18), and separated by the analytical column (Thermo scientific EASY column, 10 cm, ID75 μm, 3 μm, C18-A2). The flow rate was 300 nL/min. Two-hour gradient: 0–110 min, buffer B linear gradient from 0 to 55%; 110–115 min, buffer B linear gradient from 55 to 100%; 115–120 min, buffer B maintained at 100%.

The samples were analyzed on a Q Exactive^TM^ mass spectrometer (Thermo Fisher Scientific). Parameters were set as follows: analysis time 120 min; detection mode: positive ion; scanning range of parent ion: 300–1,800 m/z; first-order mass spectrometry resolution: 70,000 at 200 m/z; AGC (automatic gain control) target: 1e6; maximum IT (inject time): 50 ms; dynamic exclusion: 60 s. The mass charge ratio of polypeptide and polypeptide fragments was collected according to the following methods: after each full scan, 20 fragments (MS2 scan) were collected. The MS2 activation type was HCD, the isolation width was 2 m/z, the secondary mass spectrum resolution was 17,500 at 200 m/z. Normalized collision energy was 30 eV, and the underfill ratio was defined as 0.1%.

### Sequence Database Searching

Relative intensity-based label-free quantification (LFQ) was analyzed by MaxQuant version 1.5.3.17 (Max Planck Institute of Biochemistry in Martinsried, Germany) and searched against the UniProtKB *Equus asinus* database (47,825 total entries, downloaded on August 12, 2019) and *Homo sapiens* (included 20,422 series, downloaded on May 22, 2019). Proteins with *p* < 0.05 and fold change >2 or <0.5 were deemed significantly expressed between groups by *t*-test. The false discovery rate (FDR) for peptide and protein identification was set to 1%.

### Bioinformatics Analysis

Quantified MFGM samples were used to performing hierarchical clustering analysis. For this purpose, Cluster3.0 (http://bonsai.hgc.jp/~mdehoon/software/cluster/software.htm) and the Java Treeview software (http://jtreeview.sourceforge.net) were used. GO enrichment on three ontologies (biological process, molecular function, and cellular component) and KEGG pathway enrichment analyses were applied based on the Fisher' exact test, considering the whole quantified protein annotations as background dataset using DAVID (https://david.ncifcrf.gov/). Benjamini-Hochberg correction for multiple testing was further applied to adjust derived *P*-values, and only functional categories and pathways with *P*-values under a threshold of 0.05 were considered as significant.

## Results

### Quantitative Overview of Identified MFGM Proteins in DM and HM

**Figure 1:**
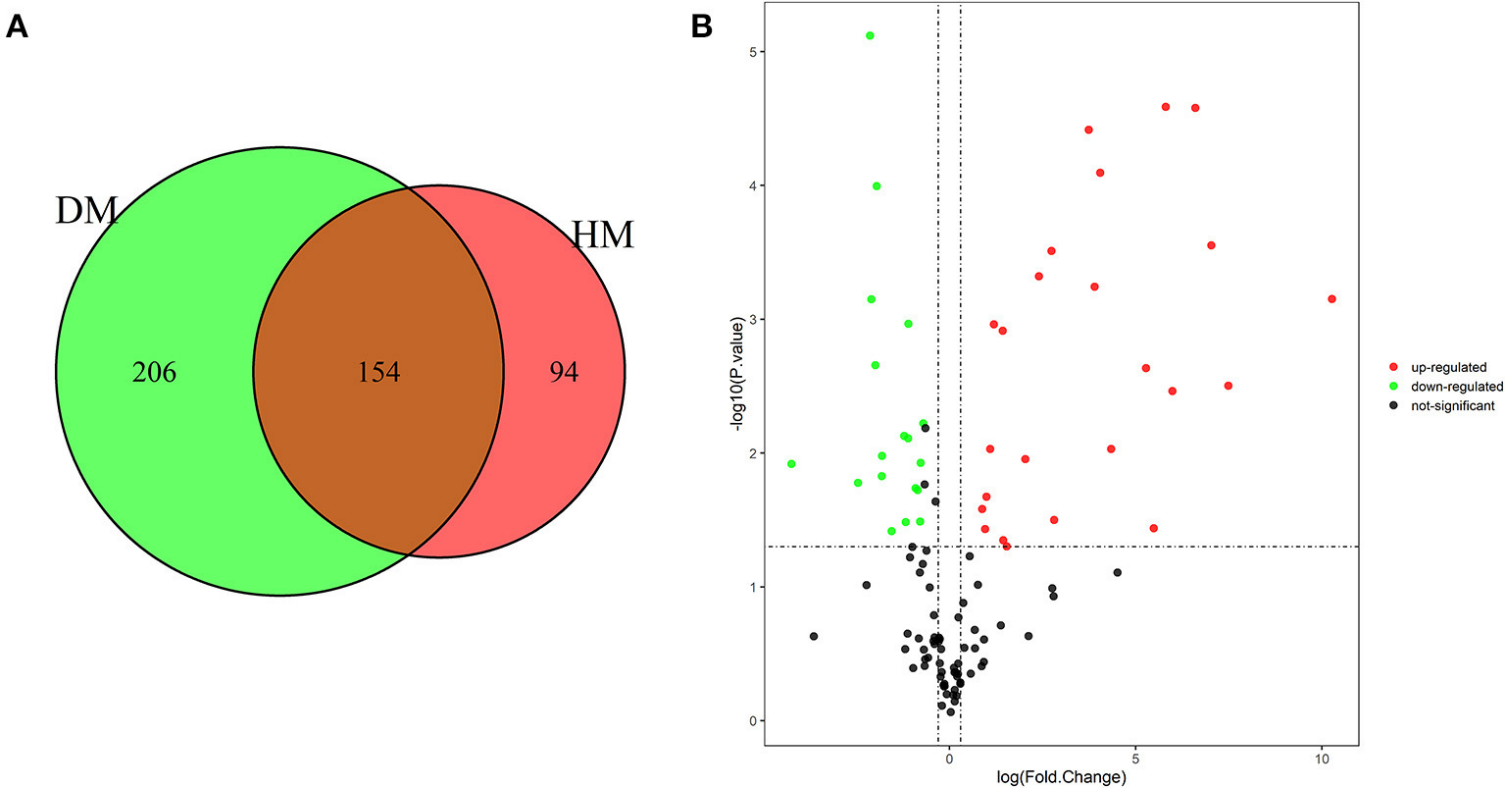
Venn diagram of MFGM proteins identified from DM and HM group **(A**); Volcano plot of proteins identified from DM and HM group **(B)**. Red dot, up-regulated proteins in DM and HM MFGM; Green dot, up-regulated proteins in DM and HM MFGM; Black dot, not significant different proteins between DM and HM. MFGM, milk fat ball membrane; DM, donkey milk; HM, human milk.

As shown in Figures 1A, 454 MFGM proteins were identified in DM and HM. Proteins having at least two replicates and fold changes >2.0 or <0.5 and *P* < 0.05 were defined as differentially expressed proteins (DEP). In addition, the proteins with at least two replicates in one group and in another group with null values were also defined as DEP. A volcano plot was used to show significant differences between the two groups based on the fold change and *P*-value (Figure 1B). Compared with MFGM proteins in HM, 204 MFGM proteins in DM were upregulated and 89 MFGM proteins were downregulated. DEP detailed information is shown in Supplementary Table 1.

### Cluster Analysis

**Figure 2:**
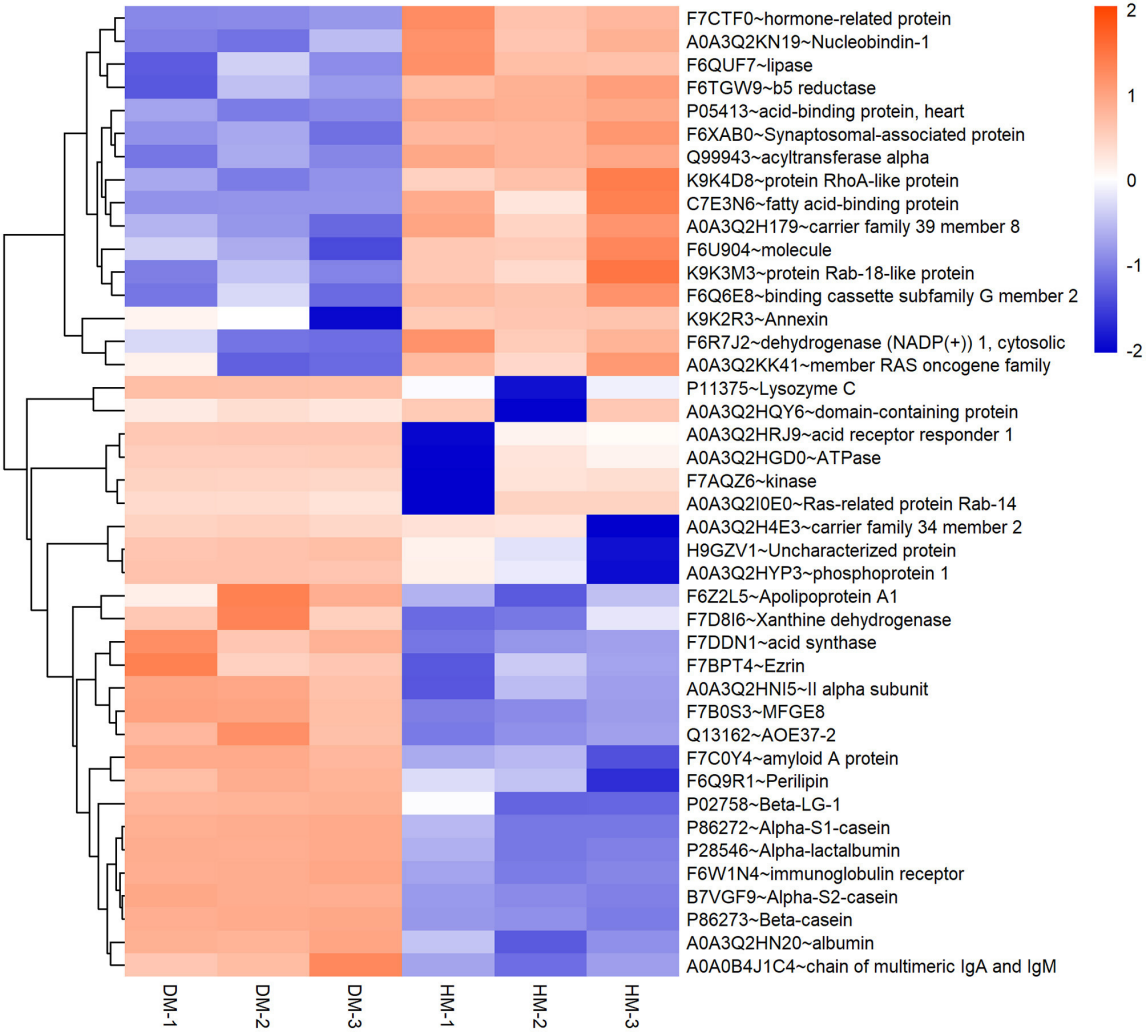
Hierarchical clustering of DEP in DM and HM group. Bar color represents a logarithmic scale from −2 to 2. DEP, differentially expressed proteins; DM, donkey milk. HM,: human milk.

Meanwhile, a hierarchical cluster analysis of MEGM proteins in DM and HM group was shown in Figure 2. The upregulated MFGM proteins in DM compared with HM mainly included alpha-s2-casein, lysozyme, alpha-s1-casein and secreted phosphoprotein 1 (SPP1), and the downregulated MFGM proteins were lipoprotein lipase, fatty acid-binding protein 3 and nucleobindin 1.

### GO Analysis of DEP in DM Compared With HM and CM

**Figure 3:**
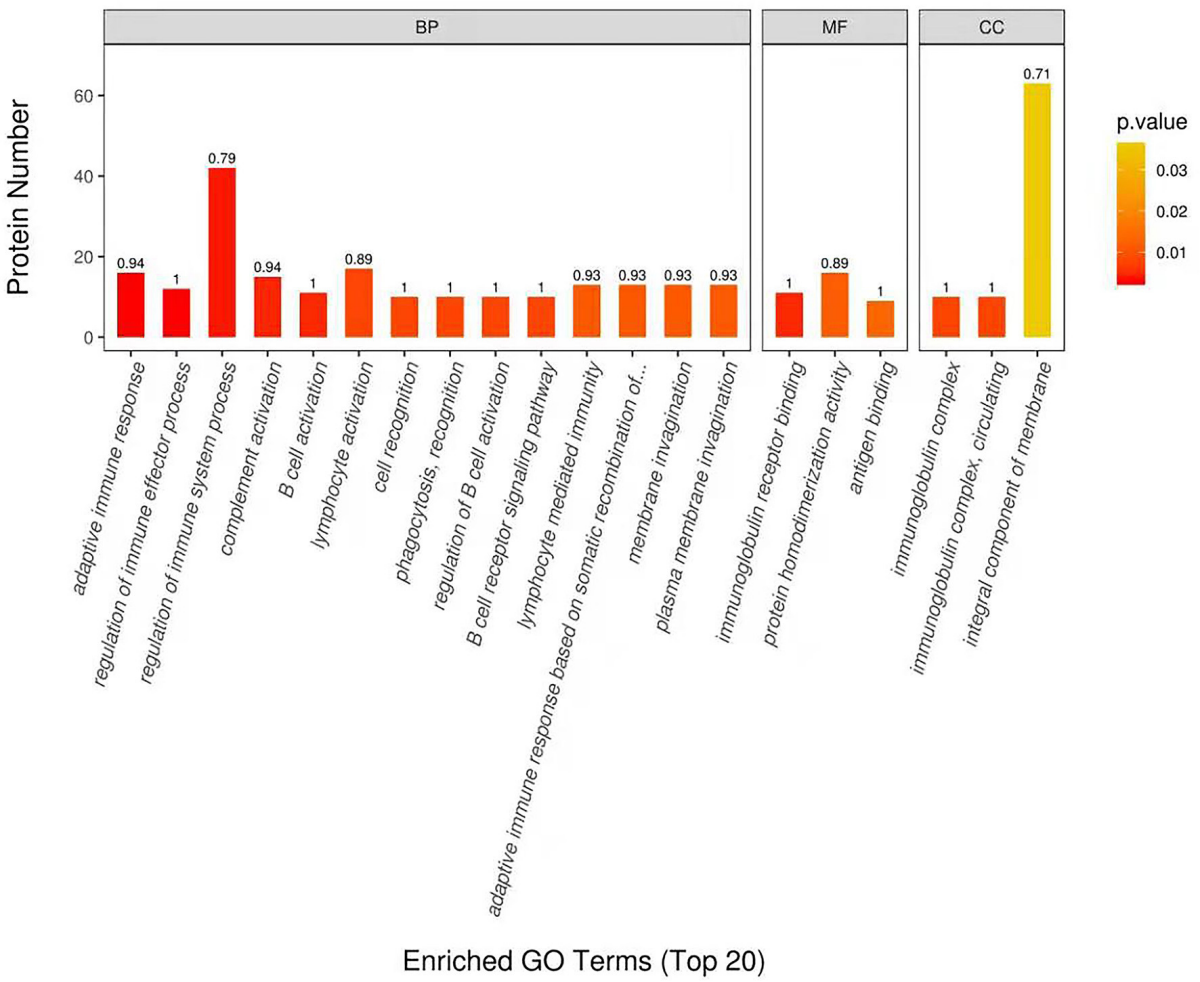
Enriched Gene Ontology (GO) Terms of the DEP in DM and HM group. GO enrichment of DEP on three categories. BP, biological processes; MF, molecular functions; CC, cellular components; DEP, differentially expressed proteins; DM, donkey milk; HM, human milk.

DEP were then classified into the GO enrichment analysis of three distinctive functional sets, cellular component, molecular function, and biological process. In terms of molecular function, DEP in DM and HM group were primarily related to immunoglobulin receptor binding, protein homodimerization activity and antigen binding (Figure 3). In the category of biological process, DEP in these two groups were mainly involved in regulation of immune system process, membrane invagination and lymphocyte activation. GO enrichment of DEP information is listed in Supplementary Table 2.

### KEGG Pathway Analysis of DEP in DM Compared With HM and CM

**Figure 4:**
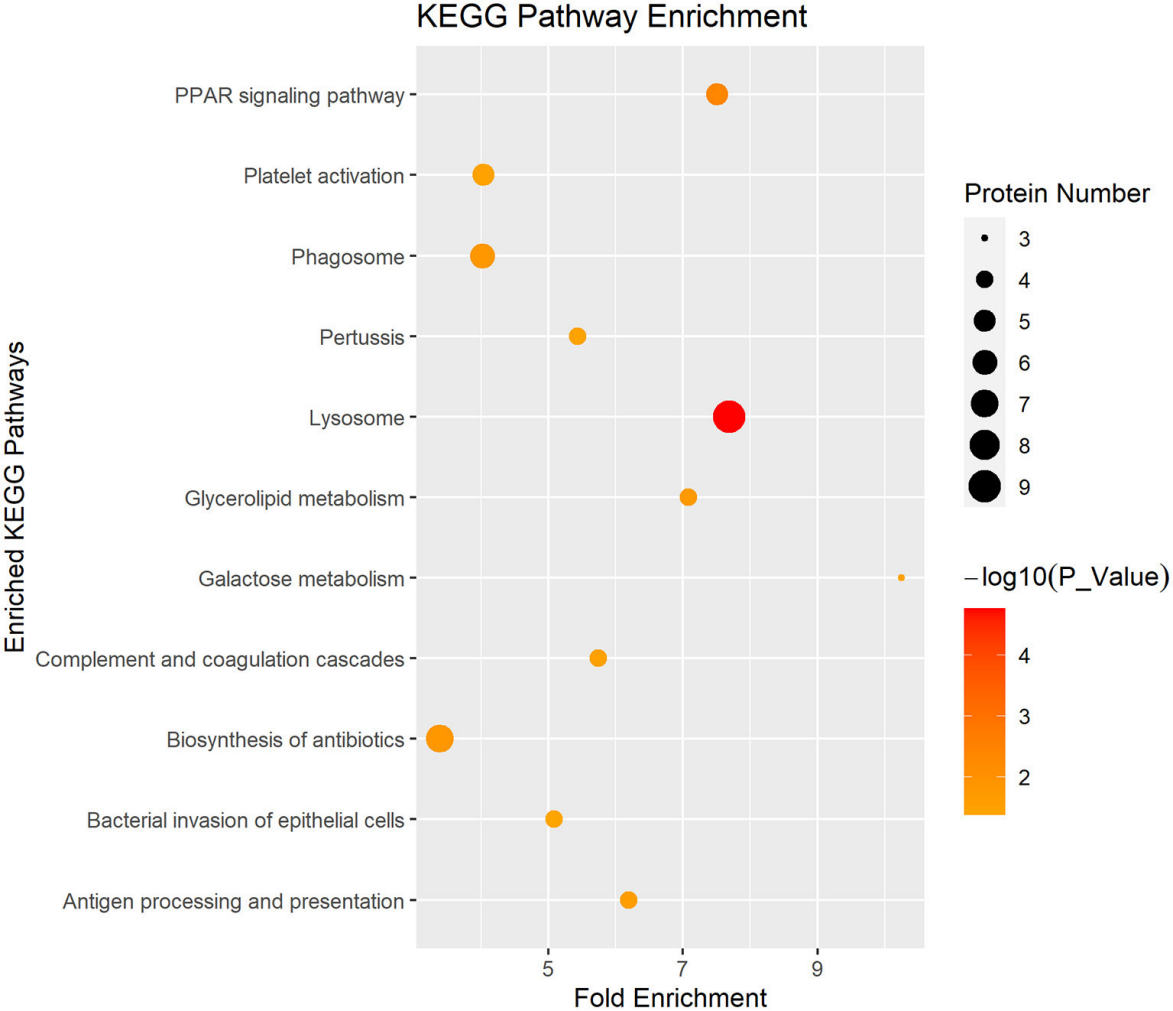
Enriched KEGG pathway analysis of the DEP in DM and HM group. KEGG, Kyoto Encyclopedia of Genes and Genomes pathways; DEP, differentially expressed proteins; DM, donkey milk; HM, human milk.

In Figure 4, the DEP were mainly involved in lysosome, galactose metabolism and PPAR signaling pathway. KEGG pathway enrichment of DEP information was listed in Table 1.

**Table 1:** Detailed information of KEGG pathway-based enrichment of the differentially expressed proteins between DM and HM.

**Pathway_ID**	**KEGG pathways**	**Protein number**	**Genes**	**Fold enrichment**	***P*-value**
ecb04142	Lysosome	9	F6YQM5, F7BPX8, F6YNH6, F6V812, F7B6D0, F7DG10, F6V3X9, F7BV85, F6VUW2	7.69	1.72E-05
ecb03320	PPAR signaling pathway	5	F7AYT5, F6Z2L5, F6RM73, F6QUF7, F6U904	7.51	4.07E-03
ecb04145	Phagosome	6	Q95M34, Q5XWB8, F7BPX8, F7DG10, F6U904, F6VUW2	4.02	1.56E-02
ecb01130	Biosynthesis of antibiotics	7	F6X8Q2, F6TL52, F6SX98, F6Y688, F6RI26, L7MRN0, F6R7J2	3.38	1.58E-02
ecb00561	Glycerolipid metabolism	4	F6TN81, F6QUF7, F7B6D0, F6RI26	7.08	1.79E-02
ecb04612	Antigen processing and presentation	4	Q9GKX8, F7BPX8, F6YNH6, F6VUW2	6.19	2.55E-02
ecb04610	Complement and coagulation cascades	4	F6USP9, F6XSF7, F6PH38, F6XGE0	5.74	3.10E-02
ecb00052	Galactose metabolism	3	F6X8Q2, F7B6D0, F6SUZ2	10.25	3.33E-02
ecb04611	Platelet activation	5	F6QMB8, F6XAB0, F7AQZ6, K9K4D8, F6PH38	4.03	3.36E-02
ecb05133	Pertussis	4	Q6TGR2, Q5XWB8, F6XSF7, K9K4D8	5.43	3.57E-02
ecb05100	Bacterial invasion of epithelial cells	4	F6VZN7, F6Y0D9, K9K4D8, F7BV85	5.08	4.22E-02

## Discussion

In this study, 293 MFGM proteins were found to be significantly different between DM and HM. These DEP were involved in regulation of immune system process, complement activation and integral component of membrane. These results may provide valuable information in the MFGM composition of DM and HM, especially for low abundant components, and expand our knowledge of different biological functions between DM and HM.

Caseins in milk exert multifunctional effects including amino acid and calcium supply (5), cellular immune functions stimulation (7, 8), and chemotactic properties (9). Studies of human, bovine, goat, and camel MFGM proteomes identified milk caseins in the MFGM fractions (2, 6, 10). In our data, four caseins (α-s1-casein, α-s2-casein, β-casein, κ-casein) were detected in DM MFGM fractions and significantly higher than that in HM. The main role of human α-s1-casein is to serve as an amino acid source to the newborns. Beyond nutritional aspects, α-s1-casein could contribute to the development of immune system. Moreover, α-s1-casein in nursed individuals gives rise to sustained specific IgG production (8). Cocco et al. found that single amino acid substitution in the specific linear epitopes of alpha s1-casein can significantly reduce the binding ability of serum IgE in patients with CM allergy (11). Donkey and cow milk alpha s1-casein share a low sequence homology, and particularly their IgE binding linear epitopes have remarkable differences in amino acid sequences (12). Moreover, Bertino et al. found that donkey alpha-s1-casein appears as both phosphorylated and glycosylated forms, but neither human nor bovine alpha-s1-caseins have been reported to be glycosylated (13). Studies have shown that in many cases milk from donkey represents a safe and alternative food in both IgE-mediated and non-IgE-mediated cow's milk protein allergy (14, 15). These differences in alpha s1-casein amino acid sequence or post-translational modifications might be related to the low allergenic properties of donkey milk. As for β-casein and kappa-casein, both of them from HM and DM are more closely related to each other than the cow counterparts (16). β-casein is the major casein constituent in human MFGM and generates smaller casein phosphopeptides upon digestion to aid the absorption of calcium (17). κ-casein is the only glycosylated in the four casein families. DM κ-casein carries a higher number of potential *O*-glycosylation than that cow milk κ-casein. Therefore, DM similar to HM may contribute to inhibit the adhesion of *Helicobacter pylori* to gastric mucosa in infant (18).

In general, β-lactoglobulin can be found in the majority of milks, but not in human's milk. However, in this study we detected β-lactoglobulin in HM. It has been found that beta-lactoglobulin can be detected in the human milk within 7 days after ingestion of milk (19). We hypothesized that the mothers who donated breast milk in this study were likely to have taken other milks before providing milk samples.

According to the GO enrichment analysis, DEP in DM vs. HM group were mainly involved immune response, such as complement activation, defense response or positive regulation of B cell activation (Supplementary Table 2). It is well-known that milk could provide large amounts of bioactive components to the infants in the critical phase of immunological immaturity. Mature breast milk could enhance B cell proliferation and antibody secretion (20). Among the DEP involved in the positive regulation of B cell activation, 9 proteins were all upregulated in DM, including immunoglobulin heavy constant gamma 3 (IGHG3) and immunoglobulin kappa constant (IGKC), which all function in B cell selection or antigen recognition (21). In addition, 49 DEP were involved in defense response, among which 32 proteins were upregulated in the DM MFGM, namely semaphorin 7A, complement 3 (C3), joining (J) chain. Semaphorin 7A (also known as CD108) plays a key role in innate immune regulation. Semaphorin 7A could induce proinflammatory cytokines production (22). Semaphorin 7A-deficient mice are defective in T cell-mediated inflammatory responses, indicating the role of semaphorin 7A in evoking inflammatory immune reactions (23). In addition to its role in the immune response, semaphorin 7A also functions as a chemoattractant and stimulates neuronal migration, which is an essential process in central nervous system development (24). The defect of neuron migration may lead to nervous system disorder (25). J chain is a small polypeptide contained in dimeric IgA and pentameric IgM, which plays an important role in the generation of secretory antibodies (26). This peptide can be produced by immunocytes of all Ig isotypes, but it becomes incorporated only into IgA and pentameric IgM (27). Moreover, the expression of J chain may be a marker of B cell clone in mucosa associated lymphoid tissue, as there is a positive correlation between the production of polymeric IgA, IgG or IgD-producing cells and J chain (28). C3 is an important part of the innate immune system. It combines with other complement proteins to form the main host mechanism for detecting and eliminating potential pathogens (29). Complement proteins contribute to the establishment of natural immune system in newborns (2). The presence of abundant immunological factors in DM MFGM proteins are helpful for the newborns to establish an immune system against microbial infection to adapt to the new environment to prevent diseases.

In this study, a solute carrier (SLC) superfamily, namely, SLC34A2, SLC36A1, SLC4A9, and SLC9A3R1, was more abundant in DM MFGM proteins. This superfamily is a major membrane transporter group that controls the uptake and excretion of nutrients, neurotransmitters, metabolites, drugs and toxins (30). SLC34A2 is a member of SLC34 family, a group of phosphate transporters, which are responsible for transporting inorganic phosphate. Phosphate is an essential nutrient for life and a key component of bone formation (31). Recently, it was concluded that SLC34A2 was responsible for the sodium-dependent component of intestinal phosphate absorption (32). SLC36A1, an amino acids transporter in small intestinal enterocytes, could regulate cell growth and sense the availability of amino acids in other cell types. SLC36A1 also could be a target for rapamycin complex 1 (TORC1) activation (33). TORC1 regulates some metabolic pathways and adapts cells to applied bioenergy and anabolic conditions (34). SLC9A3R1 is a multifunctional scaffold protein, which is involved in cell activities and affects many protein functions, including ion channels, receptors, signaling and nuclear proteins. In addition, SLC9A3R1 has potential antitumor effects in breast cancer (35). SLCs also play an important role in the function of the central nervous system. A total of 287 SLC genes were identified in the brain, especially in the barrier cells. SLCs expressed in neurons and glial cells play irreplaceable roles in maintaining brain homeostasis (36). Moreover, we also found some other abundant proteins in DM MFGM that are expressed in the brain, such as neuro plastin (NP). NP is a cell adhesion molecule rich in synaptic membranes and belongs to immunoglobulin superfamily. Np plays a role in synaptic plasticity and neurite growth (37, 38). In recent years, NP has attracted much attention because of its correlation with adolescent cortical thickness and intelligence (39). Therefore, the abovementioned functional components in DM MFGM may play a more important role in promoting the growth and learning of newborn infants.

Among these DEP, a family of apolipoproteins (Apos), namely, apolipoprotein A1 (ApoA1), ApoA2, ApoC3, ApoD, and ApoE, were significantly upregulated in DM MFGM. ApoA1 and ApoA2 are the major proteins in high-density lipoproteins (HDL) (40). ApoA1 has multiple beneficial functions, including potent antioxidant, anti-inflammatory, antiviral and antibacterial activities in blood (41, 42). Recently, ApoA1 was found in HM and DM. Kim et al. identified that ApoA1 interacts with cholesterol in HM, provides antioxidant activity and improves embryo survivability (43). In DM MFGM, ApoA1 was upregulated in colostrum compared with mature milk (2). ApoD is a glycosylated protein involved in lipid transport, food intake, inflammation, antioxidant response and development. In humans, ApoD levels rise considerably in association with aging possibly in response to accumulated damage (44). Overexpression of human ApoD could protect drosophila against various extrinsic stresses and extend its normal lifespan (45). ApoE is an important element in the lipoprotein metabolism and cholesterol transport (46). Cholesterol plays a key role in vitamin D and steroid hormones synthesis, which is critical to the development of the newborns (47). In our study, DM MFGM provides a higher level of cholesterol transporters, which may help the newborns acquire a large amount of cholesterol.

In conclusion, 204 up-regulated proteins were identified in the lipid globules of donkey milk. Through GO functional annotation and KEGG pathway enrichment analysis, we found that these upregulated proteins not only have nutritional effects, but also promote the improvement of the immune system, cognitive learning and anti-oxidation of newborns. Therefore, donkey milk might be used as a nutritional provider in infant formula. In addition, the comparison of the fat globular membrane protein between donkey milk and cow milk was missing in this study, which was a limitation of our research and will be carried out in the future.

## Conclusion

A quantitative proteomic method was used to investigate the MFGM proteins proteome in DM and HM. DEP were analyzed by multivariate statistical methods and found mainly involved in regulation of immune system process, membrane invagination and lymphocyte activation. Our findings also provided more in-depth reference for the dairy food industry and infant health.

## Data Availability Statement

The raw data supporting the conclusions of this article will be made available by the authors, without undue reservation.

## Ethics Statement

The studies involving human participants were reviewed and approved by Institutional Animal Care and Use Committee of Shangdong Agricultural University. The patients/participants provided their written informed consent to participate in this study. The animal study was reviewed and approved by Institutional Animal Care and Use Committee of Shangdong Agricultural University. Written informed consent was obtained from the owners for the participation of their animals in this study.

## Author Contributions

FZ designed the experiment and revised the manuscript. XZ completed the experiment and drafted the manuscript. BJ and CJ participated in improving the experiment. HL, LY, GJ, YW, GuaL, and GuiL participated in collecting and preparing the samples. LM helped to revise the manuscript. All authors contributed to the article and approved the submitted version.

## Conflict of Interest

XZ, CJ, HL, LY, GJ, YW, GuaL, and FZ were employed by the company Dong-E E-Jiao Co., Ltd. The remaining authors declare that the research was conducted in the absence of any commercial or financial relationships that could be construed as a potential conflict of interest.
