# Supplementary material for: Quantitative Label-Free Proteomic Analysis of Milk Fat Globule Membrane in Donkey and Human Milk
Source: Front Nutr. 2021 Jun 22;8:670099. doi: 10.3389/fnut.2021.670099 (PMC8258387; doi:10.3389/fnut.2021.670099)
Supplement: Supplementary file 1 [file Data_Sheet_1.PDF]

1.Detailed information of the differentially expressed proteins

1.1 Information for each significantly up-regulated protein (P < 0.05) identified in DM vs HM group

UniProt Accession no.	Protein Name	Gene Name	LFQ intensity						Fold change	t test p value
			DM-1	DM-2	DM-3	HM-1	HM-2	HM-3		
B7VGF9	Alpha-S2-casein	CSN1S2	7.57E+11	5.82E+11	5.40E+11	3.68E+07	1.75E+07	1.07E+07	2.89E+04	7.05E-04
P11375	Lysozyme C	LYZ	2.14E+11	1.83E+11	1.58E+11	1.47E+08		5.98E+07	1.79E+03	3.14E-03
P86272	Alpha-S1-casein	CSN1S1	3.75E+11	4.32E+11	5.01E+11	9.40E+08	1.06E+08	1.08E+08	1.13E+03	2.80E-04
P28546	Alpha-lactalbumin	LALBA	3.42E+11	3.19E+11	3.74E+11	1.01E+09	1.78E+08	2.25E+08	7.34E+02	2.63E-05
F7C0Y4	amyloid A protein	LOC100056936	6.72E+10	6.67E+10	3.91E+10	1.66E+08	2.57E+08	1.12E+07	3.99E+02	3.45E-03
P86273	Beta-casein	CSN2	4.54E+11	4.91E+11	5.32E+11	1.95E+09	1.54E+09	9.51E+08	3.33E+02	2.59E-05
H9GZV1	Uncharacterized protein		1.81E+10	2.24E+10	3.63E+10	2.06E+08	6.74E+06		2.41E+02	3.65E-02
A0A3Q2HYP3	phosphoprotein 1	SPP1	7.30E+10	7.48E+10	5.77E+10	6.72E+08	2.75E+07		1.96E+02	2.32E-03
F7B0S3	Uncharacterized protein	MFGE8	1.40E+11	1.33E+11	6.57E+10	1.10E+09	1.38E+09	1.94E+09	7.67E+01	9.33E-03
P02758	Beta-LG-1	LGB1	3.15E+11	3.36E+11	3.82E+11	1.76E+10	2.13E+08	2.37E+08	5.73E+01	8.03E-05
A0A3Q2HRJ9	acid receptor responder 1	RARRES1	3.90E+08	4.57E+08	4.49E+08		1.18E+07	5.71E+06	4.92E+01	5.71E-04
F6W1N4	immunoglobulin receptor	PIGR	9.76E+09	1.02E+10	1.15E+10	3.46E+08	1.82E+08	2.17E+08	4.22E+01	3.84E-05
F7DDN1	acid synthase	FASN	6.53E+09	2.50E+09	3.50E+09	1.82E+08	2.69E+08	3.04E+08	1.66E+01	3.16E-02
A0A3Q2HN20	albumin	ALB	5.26E+10	5.02E+10	6.40E+10	6.14E+09	1.53E+09	3.12E+09	1.55E+01	3.08E-04
A0A3Q2HGD0	ATPase	ATP2B2	4.25E+08	3.99E+08	4.45E+08		5.91E+07	1.75E+07	1.10E+01	4.79E-04
Q13162	AOE37-2	PRDX4	2.77E+08	4.52E+08	2.49E+08	3.53E+07	4.30E+07	4.93E+07	7.68E+00	1.11E-02
A0A3Q2H4E3	carrier family 34 member 2	SLC34A2	1.97E+09	2.52E+09	1.26E+09	4.67E+08	3.59E+08		4.64E+00	4.99E-02
F7BPT4	Ezrin	EZR	1.07E+09	4.96E+08	5.51E+08	1.03E+08	2.26E+08	1.72E+08	4.23E+00	4.49E-02
F6Q9R1	Perilipin	PLIN2	8.01E+09	9.59E+09	8.82E+09	3.04E+09	2.49E+09	7.79E+08	4.19E+00	1.22E-03
A0A3Q2HNI5	II alpha subunit	GANAB	1.39E+09	1.35E+09	1.11E+09	2.75E+08	4.85E+08	4.10E+08	3.30E+00	1.09E-03
A0A0B4J1C4	chain of multimeric IgA and IgM	JCHAIN	3.72E+09	4.01E+09	5.72E+09	1.64E+09	1.26E+09	1.60E+09	2.99E+00	9.34E-03
F7AQZ6	kinase	LYN	2.22E+08	2.09E+08	1.57E+08		5.87E+07	8.67E+07	2.70E+00	2.12E-02
F6Z2L5	Apolipoprotein A1	APOA1	1.40E+09	2.82E+09	2.11E+09	8.93E+08	5.89E+08	9.45E+08	2.61E+00	3.70E-02
F7D8I6	Xanthine dehydrogenase	LOC100054601	5.62E+10	8.46E+10	5.39E+10	2.15E+10	2.27E+10	3.66E+10	2.41E+00	2.62E-02

A0A3Q2I8J7	lectin domain-containing protein		6.50E+06	8.10E+06						
F7CLE3	pyrophosphatase/phosphodiesterase 3	ENPP3	8.32E+06	1.57E+07						
K9KFF1	protein (Fragment)		8.69E+06	9.23E+06						
Q9TQV1	Transferrin(Fragment)		8.73E+06	2.58E+07	1.30E+07					
K9KB26	emp24 domain-containing protein 10-like protein (Fragment)		8.76E+06	7.46E+06						
F6TBM0	17-beta-dehydrogenase 11-like protein	HSD17B11	9.78E+06	6.47E+07						
F6TL52	kinase isoenzyme 1	AK1	1.01E+07	1.05E+07						
A0A3Q2I9V2	transport protein	SFT2D2	1.03E+07	1.43E+07	1.47E+07					
A0A3Q2HV56	protein	RPL11	1.06E+07	7.73E+06						
Q13228	oxidase	SELENBP1	1.17E+07	2.76E+07						
F6PNM1	cell derived factor 4	SDF4	1.20E+07	7.67E+06	1.49E+07					
Q861L3	class I antigen (Fragment)		1.20E+07	2.44E+07						
A0A3Q2HQH1	domain-containing protein		1.26E+07	1.35E+07						
F6Y426	Nucleobindin 2	NUCB2	1.27E+07	1.26E+07	1.23E+07					
P69905	subunit alpha	HBA1	1.30E+07	2.39E+07						
P62826	nuclear protein Ran	RAN	1.30E+07	5.19E+06	4.74E+06					
K9KEF8	reticulum resident protein ERp44-like protein (Fragment)		1.32E+07	1.43E+07	1.20E+07					
L7MRN0	aminotransferase (Fragment)	GOT1	1.47E+07	1.11E+07						
P14625	GRP-94	HSP90B1	1.54E+07	1.27E+07	3.33E+07					
F7B4C0	domain-containing protein	MAL2	1.60E+07	3.16E+07	3.28E+07					
P01834	kappa constant	IGKC	1.72E+07	6.29E+06						
K9K4A9	protein Rap-2c-like protein (Fragment)		1.74E+07	2.43E+07						
A0A3Q2HIU8	fragment of IgA and IgM receptor	FCAMR	1.76E+07	2.42E+07	2.23E+07					
A8HDQ5	disintegrin-metalloprotease		1.76E+07	2.14E+07						
F7A676	aminopeptidase 3	LAP3	1.78E+07	2.95E+06	8.64E+05					
F6XSG4	ATPase	ATP2B1	1.80E+07	4.83E+07	5.39E+07					
F6RCA8	Peroxiredoxin-5	PRDX5	1.92E+07	1.17E+07	1.09E+07					

F6RHD5	phosphoribosyltransferase	APRT	1.97E+07	1.80E+07	2.32E+07					
F6YSK7		MARCKS	2.00E+07		5.85E+06					
A0A3Q2H6L1	cofactor protein	LOC100057176	2.06E+07	2.26E+07	2.36E+07					
Q7L0Q8	GTP-binding protein RhoU	RHOU	2.07E+07	3.67E+07	3.80E+07					
A0A3Q2IBB5	glycoprotein 1	GLG1	2.09E+07	2.53E+07						
F6U5V3	Transcobalamin 2	TCN2	2.13E+07	3.17E+07	3.53E+07					
H9GZL8	acidic ribosomal protein P2	RPLP2	2.15E+07	3.00E+07	3.94E+07					
A0A3Q2HWY7		LOC100060539	2.20E+07		4.66E+07					
F7B6D0	Alpha-galactosidase	GLA	2.23E+07	7.62E+07	4.19E+07					
F7C8R2	Tetraspanin	CD82	2.24E+07	2.65E+07	2.53E+07					
F6VBA3		SYTL1	2.36E+07	2.71E+07						
Q9GKX8	shock protein HSP 90-beta	HSP90AB1	2.42E+07	2.26E+07	1.57E+07					
Q15836	membrane protein 3	VAMP3	2.62E+07	1.46E+07						
F6SUZ2	UDP-glucose 4-epimerase	GALE	2.93E+07	2.88E+07						
F6Q3S6	associated domain containing 1	PRADC1	3.01E+07	3.16E+07	2.96E+07					
F6ZAQ9	trafficking 1	VTA1	3.07E+07	2.84E+07						
F6XSF7	C4a anaphylatoxin	LOC100059239	3.16E+07	2.40E+07						
A0A3Q2HSC6	Endoglin	ENG	3.19E+07	2.17E+07						
P01024	C3-beta-c	C3	3.19E+07	2.60E+07	5.79E+07					
F6SFY3	15, cell surface associated	MUC15	3.22E+07	5.12E+07	3.92E+07					
A0A3Q2H332	synthetase short chain family member 2	ACSS2	3.37E+07	2.43E+07	3.79E+07					
A0A3Q2IG74	Podocalyxin	PODXL	3.39E+07	3.07E+07	2.40E+07					
F6Q5L6	purinoceptor	P2RX7	3.42E+07	5.79E+07	3.19E+07					
F6WPD2	protein	IFI27	3.46E+07	4.31E+07	3.99E+07					
A0A3Q2IDD2	C3/C5 convertase	CFB	3.49E+07		4.72E+07					
F7BC79	Syntaxin 3	STX3	3.61E+07	3.69E+07	2.92E+07					
F6S8C6	PDZ domain containing family member 2	GIPC2	3.64E+07	3.22E+07	1.05E+07					
F6ZHQ5	intracellular channel protein	CLIC1	3.64E+07	2.28E+07	2.16E+07					
Q5T749	proline-rich protein	KPRP	3.67E+07	1.89E+07						
F6YCC0	Cystatin A	CSTA	3.77E+07	3.61E+07	3.56E+07					

A0A3Q2L2R4	domain-containing protein	LOC100051562	3.81E+07	4.64E+07	4.43E+07					
F6TLH4	carrier-associated membrane protein	SCAMP2	3.85E+07	2.99E+07	2.39E+07					
U3MI26	(Fragment)	CD105	3.87E+07	4.21E+07						
A0A3Q2GUH6	peroxidase	GPX4	3.88E+07	6.03E+07	2.35E+07					
F6YNH6		CTSB	3.97E+07	3.42E+07	3.72E+07					
K9K2D2	dehydrogenase		4.00E+07	2.95E+07						
F6XM13	Apolipoprotein D	APOD	4.06E+07	5.36E+07	2.97E+07					
F6W9B1	domain-containing protein	ST13	4.10E+07	4.80E+07	2.29E+07					
K9K4J7	chaperone 1-like protein (Fragment)		4.24E+07	4.42E+07	5.50E+07					
K9K410	phosphatase type IVA 2-like protein		4.24E+07	8.17E+07	7.15E+07					
A0A3Q2H3D5		CD81	4.25E+07	4.39E+07	4.14E+07					
F6Y3Z2	like proto-oncogene A	RALA	4.32E+07	9.83E+07	8.07E+07					
P01008	Antithrombin-III	SERPINC1	4.50E+07	1.67E+08						
A0A3Q2H198	protein	LOC111769297	4.61E+07	6.80E+07	2.33E+07					
F6TIR2	Lipocalin 2	LCN2	4.81E+07		2.72E+08					
F7BPX8	protein	CTSV	4.86E+07	6.71E+07	8.41E+07					
Q9TQW8	(Fragment)		4.87E+07	3.51E+07						
A0A3Q2LL78	S1 domain-containing protein	LOC100055297	5.20E+07	4.14E+07	1.15E+08					
F7C7V8	Semaphorin 7A	SEMA7A	5.62E+07	4.88E+07	5.02E+07					
F7AVT9	protein	LOC100071734	5.63E+07	4.89E+07	2.83E+07					
F6VUW2	Cathepsin S	CTSS	5.91E+07	8.46E+07	7.31E+07					
Q6TGR2	co-receptor MD-2		5.92E+07	5.57E+07	3.90E+07					
F6VJR6	Alpha-1B-glycoprotein	A1BG	6.05E+07	4.91E+07	7.41E+07					
F7AKG6	methylcytosine dioxygenase 3	TET3	6.05E+07	1.15E+08						
F6T0P6	vitamin D binding protein	GC	6.07E+07	6.09E+07	7.66E+07					
A0A3Q2I2H1	domain-containing protein		6.22E+07	1.02E+08	1.42E+08					
F7C450	2-HS glycoprotein	AHSG	6.42E+07	7.10E+07	9.47E+07					
A0A3Q2ICL4	protein	LOC111767442	6.48E+07	3.75E+07	3.03E+07					
F7CFK9	cell derived factor 2	SDF2	6.60E+07	5.78E+07						
Q14697	alpha-glucosidase AB	GANAB	7.27E+07		4.37E+07					
A0A3Q2HBP1	binding protein 1	HEBP1	7.50E+07	1.03E+08	8.62E+07					

F7DJZ1	domain-containing protein		7.83E+07	5.58E+07						
F6UJ33	Profilin	PFN1	7.83E+07	5.20E+07						
A0A3Q2H5J5	RAB10	RAB10	8.04E+07	6.82E+07	6.46E+07					
A0A3Q2L3I3	Clusterin	CLU	8.10E+07	8.78E+07	1.52E+08					
F7CSL8	domain-containing protein	SPI2	8.10E+07	8.47E+07	1.04E+08					
A0A3Q2KY85		APOH	8.33E+07	5.94E+07	6.92E+07					
F6TF18	BAX inhibitor motif containing 1	TMBIM1	8.85E+07	1.30E+08	1.06E+08					
F6VGG1	ribosomal protein S12	RPS12	8.97E+07	5.10E+07	2.88E+07					
A0A0A1E464	lambda light chain variable region (Fragment)	IGL	9.53E+07	1.05E+08	1.35E+08					
P0DN28	C-III	APOC3	9.87E+07	2.15E+08	2.24E+08					
F6V812	N-acetyl-alpha-glucosaminidase	NAGLU	1.00E+08	7.93E+07	5.70E+07					
F6QXW2	binding protein 1	PEBP1	1.00E+08		4.78E+07					
F6QK77	Deoxyribonuclease	DNASE1L1	1.02E+08	2.06E+08	1.49E+08					
F7DZ70	member RAS oncogene family	RAB3D	1.07E+08	1.60E+08	1.23E+08					
O97973	(Fragment)		1.11E+08	8.57E+07	1.27E+08					
A0A3Q2HRU9	binding protein	SDCBP	1.19E+08	1.05E+08	1.04E+08					
F7C603	phosphodiesterase acid like 3B	SMPDL3B	1.31E+08	2.51E+08	6.28E+07					
P01860	heavy constant gamma 3	IGHG3	1.32E+08	3.91E+07	3.20E+07					
A0A3Q2HQ25	protein	LOC100054641	1.34E+08	6.27E+07						
F6UL68	Transthyretin	TTR	1.35E+08	1.17E+08	1.08E+08					
F6SX98	aldolase	ALDOA	1.36E+08	3.42E+07	5.16E+07					
F7BV85	heavy chain	CLTC	1.36E+08	7.64E+07	7.87E+07					
A0A0A1E417	lambda light chain variable region (Fragment)	IGL	1.49E+08	1.14E+08	1.99E+08					
Q0R0D3	class I antigen	Eqca-16	1.51E+08	2.70E+08	1.24E+08					
P47710	Alpha-S1-casein	CSN1S1	1.57E+08	2.14E+07	2.32E+07					
Q5XWB8	receptor (Fragment)	CD14	1.62E+08	2.19E+08	1.84E+08					
F7C1X7	exchange regulatory cofactor NHE-RF	SLC9A3R1	1.63E+08	1.06E+08	8.58E+07					
A0A3Q2L6D8	cell death 6 interacting protein	PDCD6IP	1.65E+08	1.67E+08						

F7CXT0	b reductase 1	CYBRD1	1.68E+08	1.84E+08	2.25E+08					
F6Y688	cyclase/mutase family member	LSS	1.76E+08	2.84E+08	2.38E+08					
F6T835	Tetraspanin	TSPAN6	1.78E+08	1.10E+08	5.83E+07					
F6S3Y7	S100	S100G	1.84E+08	1.72E+08	1.61E+08					
A0A3Q2I2V3	A1 domain-containing protein	CTSD	1.92E+08	1.99E+08	2.48E+08					
A0A3Q2LRH1	carrier family 36 member 1	SLC36A1	1.94E+08	5.99E+08	3.33E+08					
A0A3Q2KP77	protein	DEFB1	2.04E+08	8.74E+07	2.84E+07					
A0A3Q2HM99	protein	LOC102148710	2.11E+08		1.50E+08					
Q863B0	Beta-2-microglobulin	b-2-m	2.12E+08	1.29E+08	9.24E+07					
F6VBP9	E	APOE	2.27E+08	3.06E+08	3.36E+08					
A0A3Q2LFM9	cytoplasmic 1 intermediate chain 1	DYNC1I1	2.44E+08	2.87E+08	2.96E+08					
A5YBL8	cis-trans isomerase	PPIB	2.59E+08	1.65E+08	1.76E+08					
F6TT70	protein	LOC100146423	2.60E+08	5.50E+08	6.84E+08					
A0A3Q2I5T2	domain-containing protein	CST3	2.66E+08	2.55E+08	3.11E+08					
F6RI26	dehydrogenase 9 family member A1	ALDH9A1	2.75E+08	1.56E+08						
P08334	A		2.84E+08	2.46E+08						
A0A3Q2ID55	carrier family 4 member 9	SLC4A9	2.97E+08	4.85E+08	6.17E+08					
F5CEP2	fat globule-EGF factor 8 splice variant (Fragment)	MFGE-8	3.19E+08	4.45E+08						
F6WG98		SLC6A14	3.21E+08	3.52E+08	3.43E+08					
M9ZUR8	S-transferase A1	GSTA1	3.28E+08	2.47E+08	2.06E+08					
A0A3Q2HWQ6	C3-beta-c	LOC100060505	3.60E+08	4.83E+07	2.51E+08					
A0A3Q2H3T6	protein	MFGE8	3.73E+08	3.13E+08	3.47E+08					
A0A3Q2IBR6	repeat containing protein X-linked	SRPX	3.93E+08	3.18E+08	2.97E+08					
C0HJR4	(Fragment)	MFGE8	3.94E+08	4.02E+08	2.81E+08					
F6USP9		PLG	3.95E+08	2.59E+08	2.24E+08					
A0A0A1E6E8	lambda light chain variable region (Fragment)	IGL	4.00E+08	1.27E+09	1.37E+09					
F7A684	protein	IFI27	4.13E+08	4.43E+08	5.55E+08					
F6X8Q2	Phosphoglucomutase 1	PGM1	4.64E+08	1.48E+08	1.22E+08					
F6SQS6	like 7A	METTL7A	4.68E+08	7.11E+08	4.34E+08					

Q865P6	Beta-defensin 1	defb1	4.69E+08	1.18E+08	1.12E+08					
F6VKB4		CSN3	4.80E+08	4.36E+08	4.68E+08					
B5BV04	Alpha-1-antitrypsin	Spi2-5	5.16E+08	4.33E+08	7.69E+08					
H9GZR2	protein		5.49E+08	3.31E+08	3.04E+08					
F6Y0D9	Cadherin-1	CDH1	5.57E+08	6.51E+08	6.16E+08					
A0A3Q2IBE3	uridylyltransferase	UGP2	5.60E+08	2.40E+08	1.97E+08					
F6RM73	Apolipoprotein A-II	APOA2	5.61E+08	4.98E+08	6.39E+08					
H9GZQ9	protein		6.07E+08	5.34E+08	1.07E+09					
A0A3Q2GVQ5	precursor	GRN	7.74E+08	6.36E+08	6.30E+08					
P19857	amyloid A protein	SAA1	8.29E+08	1.17E+09	4.92E+08					
F6PH38	beta chain	FGB	9.01E+08	2.94E+09	3.02E+09					
Q6X9W5	amyloid A protein (Fragment)	SAA	1.08E+09	6.42E+08	9.11E+08					
F7AYT5	synthetase long chain family member 4	ACSL4	1.34E+09	1.78E+09	9.13E+08					
A0A3Q2GXW2	domain-containing protein	CIDEA	1.54E+09	2.26E+09	2.08E+09					
A0A0A1E4I0	lambda light chain variable region (Fragment)	IGL	1.55E+09	8.73E+08	8.89E+08					
F6VF11	like 2	CHRDL2	1.69E+09	1.32E+09	1.48E+09					
F6W3F0	fibrocystin/polyductin	PKHD1	3.02E+09	1.12E+10	1.27E+10					
P08896	B/C		4.01E+09	3.17E+09	3.42E+09					
H9GZU8	protein		4.44E+09	2.49E+09	4.57E+09					
F7BL38	domain-containing protein	MUC1	4.54E+09	5.05E+09	3.48E+09					
F6XGE0	protein	CD55	4.86E+09	5.97E+09	3.94E+09					
F7CWC8	oxidase	LOC100147151	5.48E+09	8.88E+09	1.14E+10					
H9GZT5	protein		6.83E+09	5.38E+09	6.37E+09					
H9GZN9	protein		7.14E+09	9.70E+09	1.14E+10					
Q95M34	gamma 1 heavy chain constant region (Fragment)	IGHC1	8.81E+09	9.06E+09	8.53E+09					
P11376	C, milk isozyme	LYZ	2.03E+10	1.59E+10	1.48E+10					
A0A0A1E9A8	lambda light chain variable region (Fragment)	IGL	2.29E+10	2.38E+10	4.72E+09					
P82187		CSN3	3.74E+10	2.79E+10	1.18E+10					
K9K4B2	protein Rab-8B-like protein	RAB8B		6.38E+06	7.27E+06					
Q6UWP8		SBSN		9.15E+06	1.61E+07					

F6R942		A2M		1.15E+07	1.24E+07					
A0A3Q2L0G4	domain-containing protein	SRSF4		1.64E+07	1.20E+08					
A0A3Q2GV97		NPTN		1.98E+07	1.54E+07					
F7DG10	H+ transporting accessory protein 1	ATP6AP1		4.10E+07	3.04E+07					
F7CAC5	domain-containing protein			5.21E+08	1.52E+08					

1.2 Information for each significantly down-regulated protein (P < 0.05) identified in DM vs HM group

UniProt Accession no.	Protein Name	Gene Name	LFQ intensity						Fold change	t test p value
			DM-1	DM-2	DM-3	HM-1	HM-2	HM-3		
F6R7J2	dehydrogenase (NADP(+)) 1, cytosolic	IDH1	1.02E+08	7.32E+07	7.22E+07	1.88E+08	1.46E+08	1.62E+08	4.97E-01	5.99E-03
A0A3Q2I0E0	member RAS oncogene family	RAB14	1.64E+07	1.44E+07	9.06E+06		2.86E+07	2.92E+07	4.60E-01	1.18E-02
K9K3M3	protein Rab-18-like protein		1.14E+09	1.48E+09	1.16E+09	2.43E+09	2.22E+09	3.69E+09	4.54E-01	3.25E-02
C7E3N6	fatty acid-binding protein	FABP3	1.69E+09	1.70E+09	1.70E+09	3.98E+09	2.93E+09	4.99E+09	4.27E-01	1.89E-02
K9K4D8	protein RhoA-like protein	RHOA	2.57E+08	2.14E+08	2.35E+08	4.70E+08	5.18E+08	7.59E+08	4.04E-01	1.83E-02
F6XAB0	protein	SNAP23	7.11E+07	7.91E+07	6.03E+07	1.94E+08	1.98E+08	2.44E+08	3.31E-01	1.08E-03
F6Q6E8	binding cassette subfamily G member 2	ABCG2	5.51E+08	9.29E+08	5.15E+08	1.84E+09	1.73E+09	2.50E+09	3.29E-01	7.77E-03
A0A3Q2KK41	member RAS oncogene family	RAB1A	6.44E+08	1.95E+08	2.08E+08	1.09E+09	8.17E+08	1.49E+09	3.09E-01	3.28E-02
A0A3Q2H179	carrier family 39 member 8	SLC39A8	9.38E+07	7.93E+07	6.12E+07	2.79E+08	1.95E+08	3.17E+08	2.96E-01	7.43E-03
F6U904	molecule	CD36	9.51E+08	7.20E+08	3.35E+08	2.39E+09	2.33E+09	4.76E+09	2.11E-01	3.84E-02
F7CTF0	hormone-related protein	PTHLH	5.39E+07	5.56E+07	6.17E+07	4.76E+08	2.67E+08	3.04E+08	1.63E-01	1.05E-02
F6QUF7	lipase	LPL	7.31E+08	1.98E+09	1.10E+09	1.09E+10	6.34E+09	6.18E+09	1.63E-01	1.49E-02
Q99943	acyltransferase alpha	AGPAT1	5.76E+07	8.99E+07	6.57E+07	5.21E+08	4.48E+08	5.31E+08	1.42E-01	1.01E-04
F6TGW9	b5 reductase	CYB5R3	7.37E+07	1.94E+08	1.37E+08	8.09E+08	9.42E+08	1.21E+09	1.37E-01	2.20E-03
A0A3Q2HQY6	domain-containing protein	FOLR2	1.20E+07	3.36E+07	1.78E+07	1.64E+08		1.81E+08	1.23E-01	7.09E-04
P05413	acid-binding protein, heart	FABP3	5.18E+08	3.63E+08	4.06E+08	3.66E+09	3.42E+09	3.75E+09	1.19E-01	7.60E-06
A0A3Q2KN19	protein	NUCB1	6.91E+07	5.93E+07	1.34E+08	1.47E+09	6.71E+08	9.23E+08	8.57E-02	1.67E-02

K9K2R3	(Fragment)		9.86E+06	4.05E+06		3.51E+08	5.36E+08	5.69E+08	1.43E-02	1.21E-02
Q14390	hydrolase light chain 2	GGTLC2				1.13E+08	1.24E+08	1.47E+08		
O00481	subfamily 3 member A1	BTN3A1				2.70E+07	3.86E+07	6.16E+07		
O00754	alpha-mannosidase	MAN2B1				6.82E+07	6.52E+07	4.90E+07		
O60543	death activator CIDE-A	CIDEA				5.69E+08	5.47E+08	1.21E+09		
O95084	protease 23	PRSS23				2.23E+08	3.55E+08	2.87E+08		
P00441	dismutase [Cu-Zn]	SOD1					3.56E+07	3.11E+07		
P01833	immunoglobulin receptor	PIGR				4.86E+08	3.67E+08	5.23E+08		
P05186	phosphatase, tissue-nonspecific isozyme	ALPL				1.11E+07	8.40E+06	3.05E+07		
P05423	RNA polymerase III subunit RPC4	POLR3D				4.93E+10	4.28E+10	3.27E+10		
P07339	D	CTSD					1.07E+08	9.81E+07		
F7CK20	kinase	FGR				4.25E+07	4.47E+07	4.83E+07		
P16403	H1.2	HIST1H1C				6.70E+07	1.01E+09	6.72E+07		
A0A3Q2HUK6	domain-containing protein	HIST1H1B					1.90E+08	1.25E+07		
P22079		LPO				7.07E+07	1.18E+08			
F6RA08		STOM				1.95E+08	7.54E+08	2.64E+08		
K9KD26	aldehyde dehydrogenase-like protein (Fragment)					2.79E+07	1.73E+07	3.34E+07		
P34096	4	RNASE4				1.57E+09	1.99E+09	2.52E+09		
F6WCS1	heavy chain 9	MYH9					1.10E+08	1.13E+07		
Q9TUL9	inhibitor 3	TIMP3				1.59E+09	1.20E+09	1.18E+09		
P47989	dehydrogenase/oxidase	XDH				1.66E+09	1.49E+09	1.45E+09		
P48449	synthase	LSS				1.40E+08	9.87E+07	2.43E+08		
A0A3Q2LC76	ESCRT-III associated factor	IST1				1.85E+07	1.86E+07	2.06E+07		
P55259	secretory granule membrane major glycoprotein GP2	GP2				8.18E+08	7.11E+08	1.06E+09		
P60660	light polypeptide 6	MYL6				4.92E+06	3.88E+07			
P80511	S100-A12	S100A12				7.46E+07	1.02E+08			
P81605		DCD				1.06E+08	2.08E+07	2.73E+07		
Q08431		MFGE8				1.57E+09	6.69E+09	1.07E+10		
F6QMB8	protein subunit alpha 13	GNA13				1.13E+08	1.19E+08	1.49E+08		
K9KD98	receptor (Fragment)					3.72E+07	3.64E+07	6.10E+07		

A0A3Q2HYJ5	acyltransferase 4	GPAT4				4.28E+07	2.65E+07	4.32E+07		
K9K4C9	(Fragment)					9.62E+07	8.46E+08	1.00E+08		
Q8TBF2	F synthase	PRXL2B					3.93E+07	5.24E+07		
K9KDJ7	transport protein 81-like protein-like protein (Fragment)					3.28E+08	1.51E+09	1.45E+08		
Q92743	protease HTRA1	HTRA1				7.39E+07	1.25E+08	1.19E+08		
Q96CE8	4 L6 family member 18	TM4SF18				7.20E+06	3.14E+06	3.57E+07		
Q99541		PLIN2				8.71E+09	9.11E+09	1.59E+10		
F7CUM5	and integrin binding 1	CIB1				8.90E+07		9.79E+07		
Q9BXJ0	C1q tumor necrosis factor-related protein 5	C1QTNF5				1.87E+07	7.88E+07	9.66E+07		
Q9H1C7	and transmembrane domain-containing protein 1	CYSTM1				9.20E+07	9.05E+07	1.62E+08		
Q9HCL2	acyltransferase 1, mitochondrial	GPAM					2.53E+07	2.71E+07		
Q9NZG7		NINJ2				1.99E+08	5.26E+07	2.87E+07		
F7B3U1	protein-coupled receptor class C group 5 member B	GPRC5B				1.10E+08	1.48E+08	2.37E+08		
Q9UBS3	homolog subfamily B member 9	DNAJB9				7.66E+06	1.53E+07	1.45E+08		
Q9UNQ0	cassette sub-family G member 2	ABCG2				2.18E+09	2.49E+09	3.64E+09		
Q9Y4Z0	snRNA-associated Sm-like protein LSm4	LSM4				3.16E+10	4.04E+10	6.12E+10		
K9K9N6	domain-containing protein 1-like protein (Fragment)					3.06E+08	2.83E+08	3.38E+08		
Q9Y5G8	gamma-A5	PCDHGA5				4.14E+07	2.86E+07	4.85E+07		
F6TN81	G, endothelial type	LIPG				1.36E+07	4.93E+07	5.99E+07		
A0A3Q2GU69	v-SNARE homolog	YKT6				6.70E+07	8.38E+07	1.12E+08		
A0A3Q2GYF5	phosphatase	ALPL				1.07E+08	8.24E+07	1.81E+08		
A0A3Q2KMN3	rich secretory protein LCCL domain containing 2	CRISPLD2				1.75E+08	1.63E+08	1.80E+08		
A0A3Q2H6M5	like 2	HHIPL2				1.67E+09	1.84E+09	1.47E+09		
A0A3Q2HG76	protein subunit alpha o1	GNAO1				3.03E+07	1.75E+07	4.17E+07		
A0A3Q2HSB3		LPO				2.46E+09	2.14E+09	2.43E+09		
F7B5C4		VIM				3.25E+07	7.43E+08	6.29E+07		

F7CAB8	1, zinc-binding	AZGP1				9.36E+08	6.82E+08	7.15E+08		
A0A3Q2KJE2	cotransporter	LOC111772073				5.41E+08	4.99E+08	5.77E+08		
F6XHX6	type XVIII alpha 1 chain	COL18A1				1.87E+07	2.10E+07	9.22E+06		
F6V3X9	protein	MAN2B1				4.34E+07	6.81E+07	1.73E+07		
A0A3Q2LEZ9	binding lectin serine peptidase 1	MASP1				1.53E+07		1.12E+07		
A0A3Q2LT54	binding protein 2	STXBP2				2.77E+07	3.48E+07	2.29E+07		
K9K2N5	protein subfamily B member 9-like protein (Fragment)					2.66E+08	1.57E+08	2.76E+08		
F6SZJ2	domain containing 4	EHD4					3.16E+07	4.31E+07		
F6VZN7	homolog family member G	RHOG				1.85E+07	4.16E+07	2.47E+07		
F6WR95	oxidase	QSOX1				1.32E+09	1.33E+09	1.15E+09		
F6YQM5		PSAP				1.41E+08	6.19E+07	8.05E+07		
F6ZBH7	nucleotide exchange factor	SIL1				8.81E+07	5.98E+07	6.84E+07		
K9KB03	GDP-dissociation inhibitor 2-like protein					6.55E+06	2.24E+07			
F7BKA2	kinase	PDXK				7.58E+07	8.48E+07	2.17E+08		
F7BYW1	2	ADM2				2.48E+07	2.75E+07	5.13E+07		
F7CLX6	like EF-hand protein 1	CHP1					4.73E+07	1.53E+08		
