# Supplementary material for: Quantitative Label-Free Proteomic Analysis of Milk Fat Globule Membrane in Donkey and Human Milk
Source: Front Nutr. 2021 Jun 22;8:670099. doi: 10.3389/fnut.2021.670099 (PMC8258387; doi:10.3389/fnut.2021.670099)
Supplement: Supplementary file 2 [file Data_Sheet_2.PDF]

Detailed information of GO-based enrichment of the differentially expressed proteins

GO_ID	Term	Category	Test	Ref	TestAll	RefAll	Test_per	Ref_per	Over_Under	TestSeqs	RefSeqs	P value	FDR	richFactor
GO:0002250	adaptive immune response	P	16	17	293	473	0.05461	0.0359	over	P01024,F7CAB8,F6Q5L6,P01834,P01860,F7CAC5,H9GZN9,H9GZQ9,H9GZR2,H9GZT5,H9GZU8,H9GZV1,F6XSF7,O00481,A0A0B4J1C4,F6VUW2	P01024,F7CAB8,F6Q5L6,P01834,P01860,F6PZG1,F7CAC5,H9GZN9,H9GZQ9,H9GZR2,H9GZT5,H9GZU8,H9GZV1,F6XSF7,O00481,A0A0B4J1C4,F6VUW2	0.0029	0.99999	0.9411765
GO:0002697	regulation of immune effector process	P	12	12	293	473	0.04096	0.0254	over	P01024,F7CAB8,F6Q5L6,F6RM73,F6Z2L5,P01834,P01860,F6T835,F7AQZ6,F7CK20,Q92743,F7C7V8	P01024,F7CAB8,F6Q5L6,F6RM73,F6Z2L5,P01834,P01860,F6T835,F7AQZ6,F7CK20,Q92743,F7C7V8	0.0029	0.99999	1
GO:0002682	regulation of immune system process	P	42	53	293	473	0.14334	0.1121	over	P01024,A0A3Q2LEZ9,F7CAB8,F6Q5L6,P14625,F7AQZ6,F6RM73,F6Z2L5,K9KD98,P01834,P01860,A0A3Q2HWY7,A0A3Q2HWQ6,A0A3Q2IDD2,F6XSF7,F7CAC5,H9GZN9,H9GZQ9,H9GZR2,H9GZT5,H9GZU8,H9GZV1,O00481,F7CK20,P00441,F7C603,Q6TGR2,P01833,F6T835,Q9HCL2,F6VBP9,P05423,F7CUM5,K9K4D8,Q92743,F7BPT4,Q9GKX8,F7C7V8,F6VZN7,F6XM13,F7DG10,A0A3Q2L2R4	P01024,A0A3Q2LEZ9,F7CAB8,F6Q5L6,P06702,P14625,F7AQZ6,F6RM73,F6Z2L5,K9KD98,P01834,P01860,A0A3Q2HWY7,A0A3Q2HWQ6,A0A3Q2IDD2,F6XSF7,F6PZG1,F7CAC5,H9GZN9,H9GZQ9,H9GZR2,H9GZT5,H9GZU8,H9GZV1,O00481,F6RQM5,P23396,P63000,F7CK20,P00441,F7C603,Q6TGR2,P02788,P01833,P59998,P60709,F6T835,Q9HCL2,F6VBP9,P05423,P26583,F7CUM5,K9K4D8,Q92743,F7BPT4,Q9GKX8,F7C7V8,F6VZN7,F6VWX6,F6XM13,O77811,F7DG10,A0A3Q2L2R4	0.0036	0.99999	0.7924528
GO:0006956	complement activation	P	15	16	293	473	0.05119	0.0338	over	A0A3Q2LEZ9,P01024,P01834,P01860,F7CAC5,H9GZN9,H9GZQ9,H9GZR2,H9GZT5,H9GZU8,H9GZV1,A0A3Q2HWY7,A0A3Q2HWQ6,A0A3Q2IDD2,F6XSF7	A0A3Q2LEZ9,P01024,P01834,P01860,F6PZG1,F7CAC5,H9GZN9,H9GZQ9,H9GZR2,H9GZT5,H9GZU8,H9GZV1,A0A3Q2HWY7,A0A3Q2HWQ6,A0A3Q2IDD2,F6XSF7	0.0045	0.99999	0.9375
GO:0042113	B cell activation	P	11	11	293	473	0.03754	0.0233	over	Q9UBS3,F7AQZ6,P01834,P01860,F7CAC5,H9GZN9,H9GZQ9,H9GZR2,H9GZT5,H9GZU8,H9GZV1	Q9UBS3,F7AQZ6,P01834,P01860,F7CAC5,H9GZN9,H9GZQ9,H9GZR2,H9GZT5,H9GZU8,H9GZV1	0.0048	0.99999	1
GO:0034987	immunoglobulin receptor binding	F	11	11	293	473	0.03754	0.0233	over	F7CK20,P01834,P01860,A0A0B4J1C4,F7CAC5,H9GZN9,H9GZQ9,H9GZR2,H9GZT5,H9GZU8,H9GZV1	F7CK20,P01834,P01860,A0A0B4J1C4,F7CAC5,H9GZN9,H9GZQ9,H9GZR2,H9GZT5,H9GZU8,H9GZV1	0.0048	0.99999	1
GO:0046649	lymphocyte activation	P	17	19	293	473	0.05802	0.0402	over	K9K4D8,Q9UBS3,F7AQZ6,F7CK20,P00441,F6Q5L6,Q9HCL2,O00481,P01834,P01860,F7CAC5,H9GZN9,H9GZQ9,H9GZR2,H9GZT5,H9GZU8,H9GZV1	K9K4D8,Q9UBS3,F7AQZ6,P63000,F7CK20,P00441,F6Q5L6,P23396,Q9HCL2,O00481,P01834,P01860,F7CAC5,H9GZN9,H9GZQ9,H9GZR2,H9GZT5,H9GZU8,H9GZV1	0.0076	0.99999	0.8947368

GO:0008037	cell recognition	P	10	10	293	473	0.03413	0.0211	over	P01834,P01860,Q08431,F7CAC5,H9GZN9,H9GZQ9,H9GZR2,H9GZT5,H9GZU8,H9GZV1	P01834,P01860,Q08431,F7CAC5,H9GZN9,H9GZQ9,H9GZR2,H9GZT5,H9GZU8,H9GZV1	0.0078	0.99999	1
GO:0019814	immunoglobulin complex	C	10	10	293	473	0.03413	0.0211	over	P01834,P01860,F7CAC5,H9GZN9,H9GZQ9,H9GZR2,H9GZT5,H9GZU8,H9GZV1,A0A0B4J1C4	P01834,P01860,F7CAC5,H9GZN9,H9GZQ9,H9GZR2,H9GZT5,H9GZU8,H9GZV1,A0A0B4J1C4	0.0078	0.99999	1
GO:0050864	regulation of B cell activation	P	10	10	293	473	0.03413	0.0211	over	F7AQZ6,P01834,P01860,F7CAC5,H9GZN9,H9GZQ9,H9GZR2,H9GZT5,H9GZU8,H9GZV1	F7AQZ6,P01834,P01860,F7CAC5,H9GZN9,H9GZQ9,H9GZR2,H9GZT5,H9GZU8,H9GZV1	0.0078	0.99999	1
GO:0006910	phagocytosis, recognition	P	10	10	293	473	0.03413	0.0211	over	P01834,P01860,Q08431,F7CAC5,H9GZN9,H9GZQ9,H9GZR2,H9GZT5,H9GZU8,H9GZV1	P01834,P01860,Q08431,F7CAC5,H9GZN9,H9GZQ9,H9GZR2,H9GZT5,H9GZU8,H9GZV1	0.0078	0.99999	1
GO:0050853	B cell receptor signaling pathway	P	10	10	293	473	0.03413	0.0211	over	F7AQZ6,P01834,P01860,F7CAC5,H9GZN9,H9GZQ9,H9GZR2,H9GZT5,H9GZU8,H9GZV1	F7AQZ6,P01834,P01860,F7CAC5,H9GZN9,H9GZQ9,H9GZR2,H9GZT5,H9GZU8,H9GZV1	0.0078	0.99999	1
GO:0042571	immunoglobulin complex, circulating	C	10	10	293	473	0.03413	0.0211	over	A0A0B4J1C4,P01834,P01860,F7CAC5,H9GZN9,H9GZQ9,H9GZR2,H9GZT5,H9GZU8,H9GZV1	A0A0B4J1C4,P01834,P01860,F7CAC5,H9GZN9,H9GZQ9,H9GZR2,H9GZT5,H9GZU8,H9GZV1	0.0078	0.99999	1
GO:0010324	membrane invagination	P	13	14	293	473	0.04437	0.0296	over	P01834,P01860,Q08431,F7CAC5,H9GZN9,H9GZQ9,H9GZR2,H9GZT5,H9GZU8,H9GZV1,F6VZN7,P01024,K9K4D8	P01834,P01860,Q08431,F7CAC5,H9GZN9,H9GZQ9,H9GZR2,H9GZT5,H9GZU8,H9GZV1,P63000,F6VZN7,P01024,K9K4D8	0.0109	0.99999	0.9285714
GO:0099024	plasma membrane invagination	P	13	14	293	473	0.04437	0.0296	over	P01834,P01860,Q08431,F7CAC5,H9GZN9,H9GZQ9,H9GZR2,H9GZT5,H9GZU8,H9GZV1,F6VZN7,P01024,K9K4D8	P01834,P01860,Q08431,F7CAC5,H9GZN9,H9GZQ9,H9GZR2,H9GZT5,H9GZU8,H9GZV1,P63000,F6VZN7,P01024,K9K4D8	0.0109	0.99999	0.9285714
GO:0002449	lymphocyte mediated immunity	P	13	14	293	473	0.04437	0.0296	over	P01024,F7CAB8,F6Q5L6,P01834,P01860,F7CAC5,H9GZN9,H9GZQ9,H9GZR2,H9GZT5,H9GZU8,H9GZV1,F6XSF7	P01024,F7CAB8,F6Q5L6,P01834,P01860,F6PZG1,F7CAC5,H9GZN9,H9GZQ9,H9GZR2,H9GZT5,H9GZU8,H9GZV1,F6XSF7	0.0109	0.99999	0.9285714
GO:0002460	adaptive immune response based on somatic recombination of immune receptors built from immunoglobulin superfamily domains	P	13	14	293	473	0.04437	0.0296	over	P01024,F7CAB8,F6Q5L6,P01834,P01860,F7CAC5,H9GZN9,H9GZQ9,H9GZR2,H9GZT5,H9GZU8,H9GZV1,F6XSF7	P01024,F7CAB8,F6Q5L6,P01834,P01860,F6PZG1,F7CAC5,H9GZN9,H9GZQ9,H9GZR2,H9GZT5,H9GZU8,H9GZV1,F6XSF7	0.0109	0.99999	0.9285714
GO:0042803	protein homodimerization activity	F	16	18	293	473	0.05461	0.0381	over	O60543,P00441,Q9GKX8,F6RA08,P47989,Q13162,Q9UNQ0,A0A0B4J1C4,A0A3Q2LEZ9,F6QUF7,F6RM73,F6VBP9,F6Y0D9,F6YQM5,F7B6D0,F7BKA2	O60543,P00441,Q9GKX8,F6RA08,P47989,P49327,Q13162,Q9UNQ0,A0A0B4J1C4,A0A3Q2LEZ9,F6QUF7,F6RM73,F6VBP9,F6Y0D9,F6YQM5,F7B6D0,F7BKA2,F7E3Y7	0.0113	0.99999	0.8888889
GO:0003823	antigen binding	F	9	9	293	473	0.03072	0.019	over	P01834,P01860,F7CAC5,H9GZN9,H9GZQ9,H9GZR2,H9GZT5,H9GZU8,H9GZV1	P01834,P01860,F7CAC5,H9GZN9,H9GZQ9,H9GZR2,H9GZT5,H9GZU8,H9GZV1	0.0128	0.99999	1

GO:0050871	positive regulation of B cell activation	P	9	9	293	473	0.03072	0.019	over	P01834,P01860,F7CAC5,H9GZN9,H9GZQ9,H9GZR2,H9GZT5,H9GZU8,H9GZV1	P01834,P01860,F7CAC5,H9GZN9,H9GZQ9,H9GZR2,H9GZT5,H9GZU8,H9GZV1	0.0128	0.99999	1
GO:0050776	regulation of immune response	P	33	42	293	473	0.11263	0.0888	over	P01024,A0A3Q2LEZ9,F7CAB8,F6Q5L6,P14625,F7AQZ6,F6RM73,F6Z2L5,K9KD98,P01834,P01860,A0A3Q2HWY7,A0A3Q2HWQ6,A0A3Q2IDD2,F6XSF7,F7CAC5,H9GZN9,H9GZQ9,H9GZR2,H9GZT5,H9GZU8,H9GZV1,O00481,F7C603,Q6TGR2,P01833,F6T835,F7CK20,F6VBP9,P05423,F7BPT4,Q9GKX8,F7C7V8	P01024,A0A3Q2LEZ9,F7CAB8,F6Q5L6,P06702,P14625,F7AQZ6,F6RM73,F6Z2L5,K9KD98,P01834,P01860,A0A3Q2HWY7,A0A3Q2HWQ6,A0A3Q2IDD2,F6XSF7,F6PZG1,F7CAC5,H9GZN9,H9GZQ9,H9GZR2,H9GZT5,H9GZU8,H9GZV1,O00481,F6RQM5,P23396,F7C603,Q6TGR2,P02788,P01833,P63000,P59998,P60709,F6T835,F7CK20,F6VBP9,P05423,P26583,F7BPT4,Q9GKX8,F7C7V8	0.0133	0.99999	0.7857143
GO:0006911	phagocytosis, engulfment	P	12	13	293	473	0.04096	0.0275	over	F6VZN7,P01024,P01834,P01860,Q08431,F7CAC5,H9GZN9,H9GZQ9,H9GZR2,H9GZT5,H9GZU8,H9GZV1	P63000,F6VZN7,P01024,P01834,P01860,Q08431,F7CAC5,H9GZN9,H9GZQ9,H9GZR2,H9GZT5,H9GZU8,H9GZV1	0.0166	0.99999	0.9230769
GO:0050865	regulation of cell activation	P	15	17	293	473	0.05119	0.0359	over	F6VBP9,F7AQZ6,F7CK20,P00441,Q9HCL2,K9K4D8,P01834,P01860,F7CAC5,H9GZN9,H9GZQ9,H9GZR2,H9GZT5,H9GZU8,H9GZV1	F6VBP9,F7AQZ6,P63000,F7CK20,P00441,P23396,Q9HCL2,K9K4D8,P01834,P01860,F7CAC5,H9GZN9,H9GZQ9,H9GZR2,H9GZT5,H9GZU8,H9GZV1	0.0167	0.99999	0.8823529
GO:0072376	protein activation cascade	P	17	20	293	473	0.05802	0.0423	over	A0A3Q2LEZ9,P01024,P01834,P01860,A0A3Q2HWY7,A0A3Q2HWQ6,A0A3Q2IDD2,F6XSF7,F7CAC5,H9GZN9,H9GZQ9,H9GZR2,H9GZT5,H9GZU8,H9GZV1,F6PH38,P01008	A0A3Q2LEZ9,P01024,P01834,P01860,A0A3Q2HWY7,A0A3Q2HWQ6,A0A3Q2IDD2,F6XSF7,F6PZG1,F7CAC5,H9GZN9,H9GZQ9,H9GZR2,H9GZT5,H9GZU8,H9GZV1,A0A3Q2HTG2,F6PH38,P01008,F7CYR1	0.022	0.99999	0.85
GO:0002684	positive regulation of immune system process	P	31	40	293	473	0.1058	0.0846	over	P01024,A0A3Q2LEZ9,F7CAB8,F6Q5L6,P14625,F7AQZ6,K9KD98,P01834,P01860,A0A3Q2HWY7,A0A3Q2HWQ6,A0A3Q2IDD2,F6XSF7,F7CAC5,H9GZN9,H9GZQ9,H9GZR2,H9GZT5,H9GZU8,H9GZV1,O00481,F7C603,Q6TGR2,F6T835,Q9HCL2,F7CK20,P05423,K9K4D8,F7BPT4,F7C7V8,F7DG10	P01024,A0A3Q2LEZ9,F7CAB8,F6Q5L6,P06702,P14625,F7AQZ6,K9KD98,P01834,P01860,A0A3Q2HWY7,A0A3Q2HWQ6,A0A3Q2IDD2,F6XSF7,F6PZG1,F7CAC5,H9GZN9,H9GZQ9,H9GZR2,H9GZT5,H9GZU8,H9GZV1,O00481,F6RQM5,P23396,P63000,F7C603,Q6TGR2,P02788,P59998,P60709,F6T835,Q9HCL2,F7CK20,P05423,P26583,K9K4D8,F7BPT4,F7C7V8,F7DG10	0.0231	0.99999	0.775
GO:0043408	regulation of MAPK cascade	P	14	16	293	473	0.04778	0.0338	over	P00441,F6Q5L6,P80511,F6VBP9,F6QXW2,F6YQM5,Q9TUL9,F7BPT4,F7C1X7,F7CUM5,F6PH38,F7C7V8,F7DG10,P47989	P00441,F6Q5L6,P80511,F6VBP9,F6QXW2,F6YQM5,P23396,Q9TUL9,F7BPT4,F7C1X7,F7CUM5,A0A3Q2HTG2,F6PH38,F7C7V8,F7DG10,P47989	0.0245	0.99999	0.875

GO:0002694	regulation of leukocyte activation	P	14	16	293	473	0.04778	0.0338	over	F7AQZ6,F7CK20,P00441,Q9HCL2,K9K4D8,P01834,P01860,F7CAC5,H9GZN9,H9GZQ9,H9GZR2,H9GZT5,H9GZU8,H9GZV1	F7AQZ6,P63000,F7CK20,P00441,P23396,Q9HCL2,K9K4D8,P01834,P01860,F7CAC5,H9GZN9,H9GZQ9,H9GZR2,H9GZT5,H9GZU8,H9GZV1	0.0245	0.99999	0.875
GO:0051249	regulation of lymphocyte activation	P	14	16	293	473	0.04778	0.0338	over	F7AQZ6,F7CK20,P00441,Q9HCL2,K9K4D8,P01834,P01860,F7CAC5,H9GZN9,H9GZQ9,H9GZR2,H9GZT5,H9GZU8,H9GZV1	F7AQZ6,P63000,F7CK20,P00441,P23396,Q9HCL2,K9K4D8,P01834,P01860,F7CAC5,H9GZN9,H9GZQ9,H9GZR2,H9GZT5,H9GZU8,H9GZV1	0.0245	0.99999	0.875
GO:0016064	immunoglobulin mediated immune response	P	11	12	293	473	0.03754	0.0254	over	P01024,P01834,P01860,F7CAC5,H9GZN9,H9GZQ9,H9GZR2,H9GZT5,H9GZU8,H9GZV1,F6XSF7	P01024,P01834,P01860,F6PZG1,F7CAC5,H9GZN9,H9GZQ9,H9GZR2,H9GZT5,H9GZU8,H9GZV1,F6XSF7	0.0253	0.99999	0.9166667
GO:0019724	B cell mediated immunity	P	11	12	293	473	0.03754	0.0254	over	P01024,P01834,P01860,F7CAC5,H9GZN9,H9GZQ9,H9GZR2,H9GZT5,H9GZU8,H9GZV1,F6XSF7	P01024,P01834,P01860,F6PZG1,F7CAC5,H9GZN9,H9GZQ9,H9GZR2,H9GZT5,H9GZU8,H9GZV1,F6XSF7	0.0253	0.99999	0.9166667
GO:0006952	defense response	P	49	67	293	473	0.16724	0.1416	over	P01024,A0A3Q2LEZ9,P14625,A0A3Q2KP77,P01008,F6XAB0,K9KD98,P19857,Q6X9W5,F7C0Y4,P01834,P01860,P11375,P22079,A0A3Q2I0E0,P80511,P81605,A0A3Q2HSB3,F7CAC5,H9GZN9,H9GZQ9,H9GZR2,H9GZT5,H9GZU8,H9GZV1,Q865P6,F6Q5L6,F6XSF7,P08334,P08896,F7CK20,P11376,A0A0B4J1C4,F7C603,Q6TGR2,F6T835,F6PH38,P05423,F6VBP9,Q92743,F7C7V8,P00441,F6Z2L5,F6QUF7,Q9HCL2,Q9GKX8,Q15836,F6XM13,A0A3Q2I5T2	P20160,P01024,A0A3Q2LEZ9,P06702,P14625,O77811,P02788,A0A3Q2KP77,P26583,P01008,F6XWM5,F6XAB0,K9KD98,P19857,Q6X9W5,Q9N0Y1,F7BFJ1,F7C0Y4,P00709,P01834,P01860,P11375,P22079,A0A3Q2I0E0,P80511,P81605,A0A3Q2HSB3,F7CAC5,H9GZN9,H9GZQ9,H9GZR2,H9GZT5,H9GZU8,H9GZV1,Q865P6,P04406,P63000,F6Q5L6,F6XSF7,P08334,P08896,F7CK20,P11376,A0A0B4J1C4,F7C603,Q6TGR2,F6T835,A0A3Q2HTG2,F6PH38,A6NMB1,P05423,F6PZG1,F6ZIS4,F6VBP9,Q92743,F7C7V8,P00441,P21589,F6Z2L5,F6QUF7,Q9HCL2,Q9GKX8,P08670,Q15836,A0A3Q2IBQ7,F6XM13,A0A3Q2I5T2	0.027	0.99999	0.7313433
GO:1904950	negative regulation of establishment of protein localization	P	7	7	293	473	0.02389	0.0148	over	F6RM73,F6Z2L5,F6XM13,F7CLX6,F6VBP9,O60543,F7BPT4	F6RM73,F6Z2L5,F6XM13,F7CLX6,F6VBP9,O60543,F7BPT4	0.034	0.99999	1
GO:0051224	negative regulation of protein transport	P	7	7	293	473	0.02389	0.0148	over	F6RM73,F6Z2L5,F6XM13,F7CLX6,F6VBP9,O60543,F7BPT4	F6RM73,F6Z2L5,F6XM13,F7CLX6,F6VBP9,O60543,F7BPT4	0.034	0.99999	1

GO:0016021	integral component of membrane	C	63	89	293	473	0.21502	0.1882	over	P01833,F6RA08,Q9NZG7,Q9Y5G8,F7C8R2,A0A3Q2KJE2,F6Q5L6,F6T835,F7C7V8,Q6TGR2,F6WR95,Q15836,F6ZHQ5,O00481,A0A3Q2I9V2,F6XSG4,K9KB26,F7BC79,K9KD98,A0A3Q2HYJ5,A0A3Q2IBB5,F7B4C0,Q96CE8,Q99943,Q9H1C7,Q9HCL2,F7B3U1,Q9UNQ0,A0A3Q2GV97,F6Q6E8,A0A3Q2GU69,A0A3Q2HGD0,A0A3Q2H179,A0A3Q2H332,A0A3Q2H3D5,A0A3Q2H4E3,A0A3Q2H6L1,F6U904,A0A3Q2HIU8,A0A3Q2ID55,A0A3Q2I2V3,F6SQS6,A0A3Q2IG74,A0A3Q2LRH1,F6SFY3,F6TF18,F6TGW9,F6TLH4,F6W3F0,F6W1N4,F6WG98,F6WPD2,F6Y0D9,F7A684,F7BL38,F7CLE3,F7CXT0,F7DG10,H9GZQ9,H9GZR2,H9GZU8,Q0R0D3,Q861L3	P01833,F6RA08,F6VZD2,Q9HAS3,Q9NZG7,Q9Y5G8,F7C8R2,A0A3Q2I560,A0A3Q2KJE2,F6Q5L6,F6T835,F7C7V8,F6YG82,Q96G97,F7E293,Q6TGR2,A6NMB1,A0A3Q2H7N6,F6WR95,Q15836,F6ZHQ5,F6QHY8,O00481,O75911,A0A3Q2I9V2,O95573,F6XSG4,K9KB26,F6RNM2,F7BC79,A0A3Q2HIR5,Q53FP2,K9KD98,A0A3Q2HYJ5,A0A3Q2IBB5,F7B4C0,Q96CE8,Q99536,Q99943,Q9H1C7,Q9HCL2,F7B3U1,Q9UNQ0,A0A3Q2GV97,F6Q6E8,A0A3Q2GU69,A0A3Q2HGD0,A0A3Q2GWR2,A0A3Q2H179,F6RQM5,A0A3Q2H332,A0A3Q2H3D5,A0A3Q2H4E3,A0A3Q2H6L1,A0A3Q2H8Y8,F6U904,A0A3Q2HIU8,A0A3Q2I5A5,A0A3Q2ID55,A0A3Q2HWU4,A0A3Q2I2V3,F6SQS6,A0A3Q2IG74,A0A3Q2LRH1,F2VYZ0,F7CG05,F6SAH3,F6SFY3,F6SPH9,F6TF18,F6TGW9,F6TLH4,F6W3F0,F6W1N4,F6WG98,F6WPD2,F6Y0D9,F6ZH19,F7A684,F7BL38,F7CLE3,F7CXT0,F7DG10,H9GZQ9,H9GZR2,H9GZU8,Q0R0D3,Q861L3,Q9BDF6	0.0358	0.99999	0.7078652
GO:1902532	negative regulation of intracellular signal transduction	P	10	11	293	473	0.03413	0.0233	over	F7C1X7,F6T835,F6VBP9,F6Q5L6,F6QXW2,P47989,F7CUM5,Q9TUL9,F7BPT4,F7CLX6	F7C1X7,F6T835,F6VBP9,F6Q5L6,F6QXW2,P47989,F7CUM5,Q9TUL9,F7BPT4,F7CLX6,F7E3Y7	0.0383	0.99999	0.9090909
GO:0006958	complement activation, classical pathway	P	10	11	293	473	0.03413	0.0233	over	P01024,P01834,P01860,F7CAC5,H9GZN9,H9GZQ9,H9GZR2,H9GZT5,H9GZU8,H9GZV1	P01024,P01834,P01860,F6PZG1,F7CAC5,H9GZN9,H9GZQ9,H9GZR2,H9GZT5,H9GZU8,H9GZV1	0.0383	0.99999	0.9090909
GO:0002455	humoral immune response mediated by circulating immunoglobulin	P	10	11	293	473	0.03413	0.0233	over	P01024,P01834,P01860,F7CAC5,H9GZN9,H9GZQ9,H9GZR2,H9GZT5,H9GZU8,H9GZV1	P01024,P01834,P01860,F6PZG1,F7CAC5,H9GZN9,H9GZQ9,H9GZR2,H9GZT5,H9GZU8,H9GZV1	0.0383	0.99999	0.9090909
